# Medication Belief and Adherence among Patients with Epilepsy

**DOI:** 10.1155/2019/2806341

**Published:** 2019-04-23

**Authors:** Yirga Legesse Niriayo, Abraham Mamo, Kidu Gidey, Gebre Teklemariam Demoz

**Affiliations:** ^1^Department of Clinical Pharmacy, School of Pharmacy, College of Health Sciences, Mekelle University, Mekelle, Tigray, Ethiopia; ^2^Clinical Pharmacy and Pharmacy Practice Unit, Departments of Pharmacy, College of Health Sciences, Aksum University, Aksum, Tigray, Ethiopia

## Abstract

**Background:**

Medication adherence and belief are crucial to achieving the desired goal of therapy in epileptic patients. However, there is a lack of study regarding medication adherence and belief in our setting. Therefore, the purpose of this study was to investigate medication adherence and belief and associated factors among ambulatory patients with epilepsy.

**Method:**

A cross-sectional study was conducted on randomly selected epileptic patients at the neurologic clinic of Ayder Comprehensive Specialized Hospital, Ethiopia. Medication adherence and belief were assessed using self-reported questionnaires which were developed based on the review of different literatures. Data were analyzed using binary logistic regression analysis.

**Result:**

We included a total of 292 patients. Almost two-thirds (65.4%) of the patients were nonadherent to their medications. The most common cause of nonadherence was forgetfulness (48.7%) followed by inability to get medicine (28.8) and safety concern (23.5%). The majority (78.4%) of the patients had high medication necessity belief while 44.1% had high concern belief about the potential adverse effect of their medications. Overall, 39.4% of the patients had a negative belief toward their medications. Comorbidity (AOR: 3.51, 95% CI: 1.20-10.31), seizure encounter within the last 3 months (AOR: 5.45, 95% CI: 2.48-12.00), low medication necessity belief (AOR: 3.38, 95% CI: 1.14-10.00), high medication concern belief (AOR: 4.23, 95% CI: 2.07-8.63), and negative medication belief (AOR: 4.17, 95% CI: 1.74-10.02) were predictors of medication nonadherence.

**Conclusion:**

Majority of the epileptic patients were nonadherent to their medications, and more than one-third of the patients had a negative medication belief. Low medication necessity belief, high medication concern belief, negative medication belief, comorbidity, and seizure encounter were predictors of medication nonadherence. Therefore, healthcare providers should design educational programs to enhance the patients' believe about their medication in order to improve medication adherence and overall treatment outcome.

## 1. Introduction

Epilepsy is the second most troublesome neurologic disorder worldwide in terms of disability-adjusted life years [1]. Epilepsy is a global public health problem that affects more than 70 million people worldwide [2], and more than 85% of the global burden of epilepsy occurs in developing countries [3, 4]. In Africa, epilepsy affected about 10 million people [5]. Ethiopia is also one of the highly affected countries in Africa with an estimated prevalence of 5.2/1000 population [6]. Epilepsy is a debilitating illness that leads to neuropsychological impairment, impairment of quality of life, frequent physical injury, social stigma, poor academic performance, reduced employment rate, and shortened lifetime [6–8].

Despite the availability of effective medications, there is a huge gap in the treatment of epilepsy in developing countries, particularly in Africa owing to the poor healthcare system, illiteracy, poor health awareness, and cultural unacceptance of modern medicine [4, 9–12]. In developing countries, epilepsy-associated stigma leads to higher treatment gaps, poor treatment outcomes, and reduced quality of life in epileptic patients [13]. In Africa, the majority of people still believe that epilepsy is caused by an evil spirit that cannot be effectively treated with modern medicine [5]. Antiepileptic drugs (AEDs) are the mainstay of therapy in the management of epilepsy and can achieve seizure freedom in 70% of patients if effective regimen is followed [14]. However, epilepsy remains uncontrolled in the majority of patients with epilepsy in developing countries [15]. Nonadherence remains the leading cause of treatment failure in epilepsy [16, 17].

Medication adherence is the extent to which patients take their medications as prescribed with respect to dosage and dosage intervals throughout the treatment period [18]. Despite medication adherence is a crucial part of patient care to achieve the desired goal of treatment, more than half of the patients are nonadherent to their prescribed medicines [19, 20]. Studies have reported that the rate of adherence to AEDs ranges from 32 to 62% [16, 21–23]. Many factors including belief about medications, comorbidity, number of medications, duration of therapy, age, gender, and educational status have been associated with the rate of medication adherence [22, 23]. Medication belief is the most important predictor of medication adherence, and it was significantly associated with medication adherence in different studies [24, 25]. Nonadherence to AED leads to poor seizure control, frequent hospital admissions, increased healthcare cost, impaired quality of life, and increased risk of mortality [17, 26, 27].

Assessment of medication adherence and belief and identification of factors affecting adherence to ADEs is important to improving overall epilepsy treatment outcome. However, there is a lack of study on medication adherence and belief and associated factors in our setting. Therefore, the aim of the current study was to (i) assess medication adherence and belief and (ii) evaluate the association of medication belief and other factors with medication adherence in patients with epilepsy.

## 2. Material and Methods

### 2.1. Study Design and Study Setting

A cross-sectional study was conducted between January 2017 and April 2017 at the neurologic clinic of Ayder Comprehensive Specialized Hospital which is located in northern Ethiopia. It is the second largest public hospital in Ethiopia that provides service for about 10 million people.

#### 2.1.1. Study Population and Data Collection Procedure

Adult epileptic patients (age ≥ 18 years), patients who were taking at least one AED, and those who had been on follow-up for at least six months were included in the study. Patients who were too ill to complete the interview, those who refused to give consent, and those with incomplete medical records were excluded from the study.

A sample of 304 was calculated using a single population proportion formula assuming 50% rate of medication adherence among patients with epilepsy, 95% confidence level, 5% margin of error, and 10% of contingency for nonresponse rate. From a total of 304 subjects approached, 12 patients were excluded from the study due to unwillingness to give consent [5], incomplete medical record [4], and serious illness to complete the interview [3].

We recruited patients into the study during their appointment for medication refilling using simple random sampling technique. Information related to sociodemographics, medication adherence, and belief was retrieved by interviewing patients using the standardized questionnaire. The clinical and treatment-related characteristics were retrieved from patients' medical record using the data abstraction checklist.

The Belief about Medicines Questionnaire (BMQ) was used to assess patients' belief about their medication [28]. BMQ is a self-reported questionnaire that encompasses two five-item scales evaluating the patients' belief about the necessity of their medications for controlling their disease and their concerns about the potential adverse effects of taking medications. According to BMQ, participants were said to have high medication necessity belief if the average sum of the five-item medication necessity scale score (ranging from 5-25) is above the midpoint; otherwise, they were considered to have low medication necessity belief. Similarly, participants were said to have high concern belief if the average sum of the five-item medication concern scale score (ranging from 5-25) is above the midpoint; otherwise, they were considered to have low medication concern belief. The overall patients' belief about their medication was determined by necessity concern differential, in which the patient's belief was considered as positive when the average sum of the 5-item patient's medication necessity scale score exceeded the average 5-item medication concerns scale; otherwise, it was considered as negative [29].

Medication adherence was assessed using a self-reported questionnaire which was developed based on the review of different literatures [17, 20, 30, 31]. Patients were asked whether or not they missed or stopped a dose of their AED for any reason in the last month. We assessed adherence level in the last one-month period because of published literatures supporting the one-month recall period [17, 32, 33]. Accordingly, patients who took their AEDs appropriately according to the prescriber's instruction without missing or stopping a dose in the last month were said to be adherent, whereas patients who missed or stopped a dose of their AEDs within the last month were considered as nonadherent.

#### 2.1.2. Data Analysis

Data were analyzed using the Statistical Package for the Social Sciences (SPSS version 21.0). We checked multicollinearity to test the correlation among predictor variables using the variance inflation factor (VIF), and none was collinear. Univariable logistic regression analysis was performed to determine the association of each independent variable with medication adherence, and a further multivariable binary logistic regression model was done to identify predictors of medication adherence. A *p* value of <0.05 was considered statistically significant in all analyses.

#### 2.1.3. Ethical Approval

This study was approved by the Ethics Review Committee of the School of Pharmacy, College of Health Sciences, Mekelle University. All study participants were well informed about the objective and protocol of the study. Written informed consent was obtained from all participants. Additionally, the privacy of personal information was strictly preserved. All the methods were performed in accordance with approved institutional guidelines.

## 3. Result

### 3.1. Sociodemographic Characteristics

We included 292 patients in the final analysis of the study. Out of the total, 179 (61.3%) were males and the mean (± SD) age was 30.46 ± 10.82 years. More than half, 156 (53.4), were rural dwellers, and 14.4% were social drug users (Table 1).

### 3.2. Clinical and Treatment-Related Characteristics

Three-fourths (75.7%) of the participants had generalized epilepsy while 14.4% had focal epilepsy. Generalized tonic-clonic seizure (73.6%) was the most common type of seizure, and 39% of the patients had one or more comorbidities. Nearly two-thirds (61%) of the patients had been on treatment for five or more years, and 45.5% of the patients encountered seizure within the last 3 months. The most commonly prescribed AED was phenobarbitone (54.8%) followed by phenytoin (52.1%) (Table 2).

### 3.3. Medication Belief of the Participants

More than three-fourths (78.4%) of the patients had high necessity belief about the importance of their medication for controlling their illness while 44.1% had high concern belief about the potential adverse effect of their medications. Overall, 60.6% had a positive perception toward their AEDs (Table 3).

In our study, two-thirds (65.4%) of the patients were nonadherent to their AEDs. The most common cause for nonadherence was forgetfulness (48.7%) followed by inability to get medicine (28.8), safety concern (23.5%), and affordability problem (21.5%) (Table 4).

### 3.4. Factors Associated with Medication Adherence

Univariable logistic regression analysis was performed to compare adherent and nonadherent epileptic patients using the sociodemographic, medication belief, clinical, and treatment related characteristics. Accordingly, social drug use [crude odds ratio (COR): 2.21, 95% CI: 1.02-4.82], comorbidity [COR: 5.60, 95% CI: 3.06-10.25], number of medications ≥ 3 [COR: 2.21, 95% CI: 1.35-3.61], seizure encounter within the last 3 months [COR: 11.44, 95% CI: 5.96-21.96], low medication necessity belief [COR: 6.74, 95% CI: 2.8-16.26], high medication concern belief [COR: 7.00, 95%: 3.86-12.71], and negative medication belief [COR: 9.78, 95% CI: 4.92-19.46] were significantly associated with medication adherence (Table 5).

Variables with *p* < 0.2 in the univariable analyses were included in the multivariable logistic regression model. The full model containing all predictors was statistically significant (chi-square = 137.207, df = 12, *P* < 0.001). Comorbidity (AOR: 3.51, 95% CI: 1.20-10.31), seizure encounter within the last 3 months (AOR: 5.45, 95% CI: 2.48-12.00), low medication necessity belief (AOR: 3.38, 95% CI: 1.14-10.00), high medication concern belief (AOR: 4.23, 95% CI: 2.07-8.63), and negative medication belief (AOR: 4.17, 95% CI: 1.74-10.02) were found to be predictors of medication nonadherence (Table 5).

## 4. Discussion

Adherence to antiepileptic drugs is crucial to improving seizure control and overall treatment outcome in epileptic patients. However, maintaining good adherence to antiepileptic drugs remained the most important challenge in the globe, particularly in developing countries [18, 34]. Assessment of medication adherence and its contributing factors is helpful to designing programs for future intervention. Therefore, our study investigated the rate of nonadherence and its contributing factors among epileptic patients.

Studies have shown that most of the patients with epilepsy in developing countries are not treated or not appropriately treated [11, 35, 36]. In line with other similar studies [22, 23, 37, 38], majority (65.4%) of the patients were nonadherent to their AED in our study. In contrast, the rate of adherence in our study was lower than those in studies done in China [39] and Germany [40]. The reason for this variance could be the poor perception of patients toward their illnesses and medication in developing countries including Ethiopia. In addition, it could be attributed to the difference in methods of adherence assessment.

Forgetfulness was the major reason for nonadherence in our study which is also supported by other similar studies [22, 38, 41]. Next to forgetfulness, the inability to obtain medication and safety concern were common causes of nonadherence. A similar finding was also reported in another similar study [21].

In our study, majority of patients had a positive belief about their medication which is comparable with a Saudi Arabian study [21]. Our study reported that majority of the patients had a high medication necessity belief about their AEDs while over one-third of the patients had high concern belief about the potential adverse effects of their medications which is similar with a United Kingdom study [25].

Medication belief was found to be an important predictor of medication adherence across different studies [24, 42]. Consistent with these studies, belief about medication was significantly associated with the rate of medication adherence in our study. In agreement to other studies [21, 25, 38, 43], patients with low necessity belief, high concern belief, and negative medication belief toward their AEDs were more likely to be nonadherent to their medications in this study. Therefore, more effort needs to be done to improve the awareness of patients about the importance of their medication in order to improve medication adherence.

In the present study, epileptic patients with comorbidity were less likely to be adherent to their medications which is also augmented by another similar study [22]. This could be explained that patients with comorbidity are more likely to have multiple drugs. Thus, they could be reluctant to take all drugs properly. Moreover, the risk of adverse drug events is increased in patients with comorbidities which can also negatively affect the adherence rate.

Several studies revealed that nonadherence to AEDs was significantly associated with poor seizure control [16, 17, 44]. Likewise, patients with uncontrolled seizure were more likely to be nonadherent to their AEDs in the present study. This finding suggests that healthcare providers should design educational programs to enhance medication adherence so as to improve epilepsy treatment outcome.

Our study has some limitations. Our study may not provide adequate evidence regarding the cause-effect relationship of medication adherence and its risk factors due to the inherent characteristics of the cross-sectional study. In addition, as medication adherence and belief were assessed using self-reported data, patients may have underestimated these undesirable activities due to self-report concerns.

## 5. Conclusion

Majority of the epileptic patients were nonadherent to their medications, and forgetfulness was the common cause of nonadherence in this study. More than one-third of the patients had a negative belief about their medications. Low medication necessity belief, high medication concern belief, negative medication belief, comorbidity, and seizure encounter within the last 3 months were significantly associated with medication nonadherence. Therefore, healthcare providers should design educational programs to enhance the patients' belief about their medication in order to improve medication adherence and overall treatment outcome. Moreover, more emphasis should be given to patients with comorbidity and poor seizure control.

## Figures and Tables

**Table 1:** Sociodemographic characteristics of epileptic patients (*n* = 292).

Characteristics	*n* (%)
Gender	
Male	179 (61.3)
Female	113 (38.7)
Age in years	
18-30	172 (58.9)
31-60	112 (38.4)
>60	8 (2.7)
Marital status	
Married	81 (27.7)
Single	172 (58.9)
Divorce	24 (8.2)
Widowed	15 (5.1)
Residence	
Rural	156 (53.4)
Urban	136 (46.6)
Educational level	
Unable to write and read	50 (17.1)
Primary school	100 (34.2)
Secondary school	124 (42.5)
Tertiary school	18 (6.2)
Employment status	
Employed	27 (27.9)
Unemployed	207 (70.9)
Social drug use	
No	250 (85.6)
Yes	42 (14.4)
Monthly income	315 (92.7)
≤1500	152 (52.1)
>1500	140 (47.9)

**Table 2:** Clinical and treatment-related characteristics of epileptic patients (*n* = 292).

Characteristics	*n* (%)
Comorbidity	
No	178 (61.0)
Yes	114 (39)
Major comorbidities	
Psychiatric disorder	49 (16.8)
Migraine headache	25 (8.5)
Cardiovascular disorder	24 (8.2)
Respiratory disorders	16 (5.5)
Type of epilepsy	
Generalized epilepsy	221 (75.7)
Focal epilepsy	42 (14.4)
Unknown	29 (9.9)
Type of seizure	
GTC	215 (73.6)
Focal seizure	42 (14.4)
Absence seizure	6 (2.1)
Unclassified seizure	29 (9.9)
Duration of treatment	
<5	114 (39)
≥5	178 (61)
Commonly used medications	
Phenobarbitone	160 (54.8)
Phenytoin	152 (52.1)
Carbamazepine	90 (30.8)
Valproate	89 (30.5)
Seizure encounter in the last three months	
No	159 (54.5)
Yes	133 (45.5)
Number of medicines per patient	
<3 medication	142 (48.6)
≥3 medication	150 (51.4)

**Table 3:** Medication belief of the participants (*n* = 292).

Variables	*n* (%)
Overall belief about medication	
Negative belief	115 (39.4)
Positive belief	177 (60.6)
Medication necessity belief	
High necessity belief	229 (78.4)
Low necessity belief	63 (21.6)
Medication concern belief	
High concern belief	129 (44.2)
Low concern belief	163 (55.8)

**Table 4:** Adherence to AEDs and reasons for nonadherence (*n* = 292).

Characteristics	Number (%)
Medication adherence	
Adherent	101 (34.6)
Nonadherent	191 (65.4)
Reasons for nonadherence	
Forgetfulness	93 (48.7)
Inability to get medicine	49 (25.7)
Safety concern	49 (25.7)
Cost	41 (21.5)
Ran out of pills	23 (12)
Lack of hope	16 (8.4)
Not taking the medication during recovery	16 (8.4)

**Table 5:** Factors associated with medication adherence among epileptic patients (*n* = 292).

Predictors	Medication adherence		COR (95% CI)	*p* value	AOR (95% CI)	*p* value
	Adherent, *n* (%)	Nonadherent, *n* (%)				
Age in years						
18-30	60 (59.4)	112 (58.6)	1	1	1	1
31-60	36 (35.6)	76 (39.8)	0.99 (0.60-1.64)	0.971	1.83 (0.91-3.69)	0.090
>60	5 (5.0)	3 (1.6)	0.305 (0.07-1.32)	0.113	0.62 (0.07-5.55)	0.671
Social drug use						
No	92 (91.1)	157 (82.2)	1	1	1	1
Yes	9 (8.9)	34 (17.8)	2.21 (1.02-4.82)	0.045	0.79 (0.26-2.40)	0.672
Comorbidity						
No	85 (84.2)	93 (48.7)	1	1	1	1
Yes	16 (15.8)	98 (51.3)	5.60 (3.06-10.25)	<0.001	3.51 (1.20-10.31)	0.022
Types of seizure						
GTC	72 (71.3)	143 (74.9)	1	1	1	1
Focal seizure	14 (13.9)	28 (14.7)	1.85 (0.85-4.05)	0.122	0.43 (0.16-1.16)	0.096
Absence seizure	1 (1.0)	5 (2.6)	1.87 (0.71-4.93)	0.208	0.72 (0.04-13.54)	0.823
Unclassified seizure	14 (13.9)	28 (14.7)	4.67 (0.48-45.04)	0.183	0.57 (0.21-1.55)	0.272
Number of medications						
<3	62 (61.4)	80 (41.9)	1	1	1	1
≥3	39 (38.6)	111 (58.1)	2.21 (1.35-3.61)	0.002	0.53 (0.21-1.36)	0.185
Seizure encounter						
No	88 (87.1)	71 (37.2)				
Yes	13 (12.9)	120 (62.8)	11.44 (5.96-21.96)	<0.001	5.45 (2.48-12.00)	<0.001
Medication necessity belief						
High necessity	91 (90.1)	138 (72.3)	1	1	1	1
Low necessity	10 (9.9)	53 (27.7)	6.74 (2.80-16.26)	<0.001	3.38 (1.14-10.00)	0.028
Medication concern belief						
Strong concern	17 (16.8)	112 (58.6)	7.00 (3.86-12.71)	<0.001	4.23 (2.07-8.63)	<0.001
Low concern	84 (83.2)	79 (41.4)	1	1	1	1
Over all medication belief						
Negative belief	11 (10.9)	104 (54.5)	9.78 (4.92-19.46)	<0.001	4.17 (1.74-10.02)	0.001
Positive belief	90 (89.1)	87 (45.5)	1	1	1	1

COR: crude odds ratio; AOD: adjusted odds ratio; CI: confidence interval; GTC: generalized tonic seizure.

## Data Availability

The dataset used to support the findings of this study is available from the corresponding author upon request.
